# Tissue-specific expression of IgG receptors by human macrophages *ex vivo*

**DOI:** 10.1371/journal.pone.0223264

**Published:** 2019-10-15

**Authors:** Christine W. Bruggeman, Julia Houtzager, Barbara Dierdorp, Jesper Kers, Steven T. Pals, René Lutter, Thomas van Gulik, Joke M. M. den Haan, Timo K. van den Berg, Robin van Bruggen, Taco W. Kuijpers

**Affiliations:** 1 Department of Blood Cell Research, Sanquin Research and Landsteiner Laboratory, Academic Medical Center (AMC), University of Amsterdam, Amsterdam, The Netherlands; 2 Department of Experimental Surgery, Academic Medical Center (AMC), University of Amsterdam, Amsterdam, The Netherlands; 3 Department of Experimental Immunology, Academic Medical Center (AMC), University of Amsterdam, Amsterdam, The Netherlands; 4 Department of Pathology, Academic Medical Center (AMC), University of Amsterdam, Amsterdam, The Netherlands; 5 Van 't Hoff Institute for Molecular Sciences, University of Amsterdam, Amsterdam, The Netherlands; 6 Department of Molecular Cell Biology and Immunology, VU University Medical Center, Amsterdam, The Netherlands; 7 Emma Children's Hospital, Academic Medical Center (AMC), University of Amsterdam, Amsterdam, The Netherlands; Institut Cochin, FRANCE

## Abstract

Recently it was discovered that tissue-resident macrophages derive from embryonic precursors, not only from peripheral blood monocytes, and maintain themselves by self-renewal. Most *in-vitro* studies on macrophage biology make use of *in-vitro* cultured human monocyte-derived macrophages. Phagocytosis of IgG-opsonized particles by tissue-resident macrophages takes place via interaction with IgG receptors, the Fc-gamma receptors (FcγRs). We investigated the FcγR expression on macrophages both *in-vivo* and *ex-vivo* from different human tissues. Upon isolation of primary human macrophages from bone marrow, spleen, liver and lung, we observed that macrophages from all studied tissues expressed high levels of FcγRIII, which was in direct contrast with the low expression on blood monocyte-derived macrophages. Expression levels of FcγRI were highly variable, with bone marrow macrophages showing the lowest and alveolar macrophages the highest expression. Kupffer cells in the liver were the only tissue-resident macrophages that expressed the inhibitory IgG receptor, FcγRIIB. This inhibitory receptor was also found to be expressed by sinusoidal endothelial cells in the liver. In sum, our immunofluorescence data combined with *ex-vivo* stainings of isolated macrophages indicated that tissue-resident macrophages are remarkably unique and different from monocyte-derived macrophages in their phenotypic expression of IgG receptors. Tissue macrophages show distinct tissue-specific FcγR expression patterns.

## Introduction

Since the 1960s the dogma has been that the homeostasis of tissue-resident macrophages relies on the constant recruitment of blood monocytes [1]. Because of this assumption, most functional studies on macrophages have been performed on macrophages that have been *in vitro* cultured from peripheral blood monocytes. These monocyte-derived macrophages (MDMs) can be cultured with granulocyte macrophage-colony stimulating factor (GM-CSF) or macrophage-colony stimulating factor (M-CSF), generating the so-called M1 and M2 or pro-inflammatory and anti-inflammatory macrophages [2,3].

Recently, studies in rodents have shown that adult tissue macrophages are primarily derived from embryonic precursors that seed the tissues prior to birth, not from blood monocytes. These tissue-resident macrophages play a central role in homeostasis and maintain themselves by self-renewal [4–9]. Lung macrophages in the alveoli develop shortly after birth from hematopoietic stem cell-derived fetal monocytes and are not replaced by cells that derive from peripheral blood monocytes [10]. After birth, blood monocytes may indeed migrate into the tissues and differentiate into macrophages in case of inflammation [6]. Tissue-resident macrophages in the intestine and the skin are considered to be largely derived from blood monocytes, which can be explained by the fact that these sites are constantly exposed to the environment being subject to a low grade of mild inflammation. These intestinal MDMs contribute to homeostasis by having a high phagocytic and bactericidal activity, but they do not, in contrast to blood monocytes and other tissue macrophages, release proinflammatory cytokines upon phagocytosis [11–13].

Macrophages play an important role in the phagocytosis of antibody-opsonized particles, a process that takes place via interaction with Fc-gamma receptors (FcγRs). These FcγRs, expressed by macrophages and other immune cells, recognize the constant region (Fc domain) of immunoglobulin G (IgG). The family of FcγRs consists of the high-affinity receptor FcγRI and the low-affinity receptors FcγRII and FcγRIII. All FcγRs, except for FcγRIIB and FcγRIIIB, are activating receptors because they contain an immunotyrosine-based activating motif (ITAM) or associate with the common ITAM-containing γ-chain [14]. MDMs that are cultured in the presence of GM-CSF or M-CSF have a distinctive FcγR expression pattern. GM-CSF macrophages, the so-called pro-inflammatory macrophages, mainly express FcγRI and FcγRIIIA, whereas M-CSF macrophages, the anti-inflammatory macrophages, predominantly express FcγRIIA [2,3]. Because macrophages are highly heterogeneous due to their origin and more in particular due to their tissue environment [10,15,16], we studied primary human macrophages in different tissues.

A detailed comparison of the FcγR expression patterns of macrophages from human tissues has not been available to date. For this reason, MDMs and different tissue-resident macrophages were compared by immunofluorescence stainings on tissue sections, and flow cytometry following isolation of macrophages from human bone marrow, spleen, liver and lung.

## Methods

### Human subjects

Peripheral blood from healthy volunteers was obtained in heparinized tubes. Informed consent was obtained from all volunteers. Spleen tissue was from organ transplant donors, obtained as part of the surgical procedure for HLA typing, as previously described [17,18]. Bone marrow was aspirated from the sternum of patients that underwent surgery for unrelated reasons and stored in heparinized tubes. Their hematopoietic compartment was considered to be healthy. Informed consent was obtained from all individuals. Alveolar macrophages were obtained by bronchoalveolar lavage (BAL) from patients suspected of COPD or sarcoidosis for diagnostic purposes. In case there was an excess of BAL fluid, cells were used for research in the present study. The alveolar macrophages may be in a more inflamed state than cells from completely healthy individuals. Pieces of lung tissue were obtained as part of surgical procedures and thereafter anonymized. Liver samples were from patients that underwent partial hepatectomy. The representative tissue samples were obtained from organ sections that were not involved in the primary disease for which surgery had been originally planned. Materials were collected as part of the diagnostic work-up and anonymously provided by the responsible pathologist (resident), in accordance with the Dutch law regarding the use of rest material for research purposes. The study was approved by the Medical Ethics Committee of the Academic Medical Center and Sanquin in Amsterdam and was performed in accordance with the Declaration of Helsinki.

### Immunofluorescence

Small pieces of human spleen, liver or lung were embedded in Tissue Tek, frozen in liquid nitrogen vapor and stored at -80°C. Cryostat sections of 7 μm thickness were cut, fixed in acetone for 10 minutes and rehydrated in PBS. The sections were blocked with 5% human serum (Brocacef, Amsterdam, the Netherlands) in PBS for 15 minutes prior to 30 minutes staining with the following antibodies: anti-CD68 (clone Y1/82A, BD BioSciences), anti-VE-Cadherin (clone 55-7H1, BD BioSciences), anti-CD163 (clone MAC2-158, Trillium Diagnostics), anti-CD19 (clone HIB19, BioLegend), anti-CD64 (clone 10.1, BioLegend), anti-CD32a,b,c (clone AT10, Serotec), anti-CD32a (clone IV.3, SanBio), anti-CD32b,c (clone 2B6, a generous gift from MacroGenics, Rockville, MD) and anti-CD16 (clone 3G8, BioLegend). After washing with PBS, the sections were embedded in mowiol (Calbiochem). Isotype-matched antibodies were used as negative controls, i.e. the detection limit was increased until the level was reached at which the isotype control did not show any fluorescent signal anymore [18]. Stainings were analyzed using a DM6000 Leica immunofluorescence microscope or Leica SP8 confocal microscope.

### Cell isolation

Splenocytes were isolated as previously described [18]. In short, a single cell suspension of splenocytes was prepared by injecting a piece of spleen with a collagenase buffer, containing DNAse, aggrastat and glucose. After filtering the suspension, erythrocytes were removed by incubating with an ice-cold lysis buffer. Bone marrow was processed by lysing the erythrocytes twice with an ice-cold isotonic ammonium chloride lysis buffer, for five minutes. Cells from BAL fluid were spun down by centrifuging for 10 minutes at 267g, 4°C. Cells were subsequently washed in PBS containing 0.5% bovine serum albumin (BSA) and 0.2 ppm K-EDTA and used for further analysis. To obtain a single cell suspension of liver cells, cells were isolated as previously described in detail [19]. In short, a piece of liver tissue was rinsed with perfusion solution to flush out the blood, and subsequently incubated with a collagenase buffer. The non-parenchymal cells were separated from the hepatocytes by low-speed centrifugation at gradually increasing rates. The non-parenchymal cells, including Kupffer cells, were used for further analysis.

All samples were processed on the day of collection, except for spleen tissue which was usually one day old. Cytospins were made and stained with May-Grünwald Giemsa for morphological analysis. To analyze the expression pattern of FcγRs, cells were analyzed by flow cytometry (see below).

### Monocyte isolation and differentiation into monocyte-derived macrophages

Monocytes were isolated from peripheral blood mononuclear cells (PBMCs) and cultured into MDMs, essentially as described previously [2,3]. In short, monocytes were isolated by CD14 magnetic-activated cell separation (MACS) isolation kit (Miltenyi Biotec), according to manufacturer’s description. The isolated monocytes were then cultured for nine days in IMDM, supplemented with 10% FCS, L-glutamine and antibiotics, containing 10 ng/mL granulocyte macrophage-colony stimulating factor (GM-CSF) or 50 ng/mL macrophage-colony stimulating factor (M-CSF).

CD16^pos^ and CD16^neg^ monocytes were isolated by a two-step MACS isolation. First, platelet rich plasma was removed, after which monocytes were isolated from PBMCs by a negative isolation using the Pan monocyte isolation kit (Miltenyi Biotec), according to manufacturer’s description. Second, a positive isolation using the CD16 MACS isolation kit (Miltenyi Biotec) was used to separate the CD16^pos^ and CD16^neg^ monocytes. The cells were cultured in supplemented medium as described above.

### Phenotyping and genotyping

Cells isolated from the various tissues were first gated based on canonical forward-sideward scatter and subsequently on autofluorescence, in a channel for which no staining was added. Macrophages were subsequently gated based on CD163 expression, using PE-labeled anti-CD163 (clone MAC2-158; Trillium Diagnostics), as we have previously published in detail [18]. For determining FcγR surface expression the following antibodies were used: FITC-labeled anti-CD64 (clone 10.1; BD Pharmingen), anti-CD32a,b,c (clone AT10; AbD Serotec), anti-CD32a (clone IV.3; Stem Cell Technologies), anti-CD32b,c (clone 2B6) and anti-CD16 (clone 3G8; BD Pharmingen). Stainings were performed in PBS containing 1% human serum albumin and 0.01% sodium azide. Proper FITC-labeled isotype controls were taken along. Samples were measured on a FACS CANTO II (BD BioSciences). We adhered to the guidelines for the use of flow cytometry and cell sorting in immunological studies.

All individuals were genotyped for the *FCGR2/3* locus. Genotyping was performed as previously described [20–22].

### Statistical analysis

Statistical analysis was performed using GraphPad Prism 7.04. For comparison of FcγR expression levels between macrophages from the various tissues, Kruskal-Wallis tests were used, correcting for multiple comparisons using Dunn’s.

## Results

### Immunofluorescence of tissue sections

**Fig 1:**
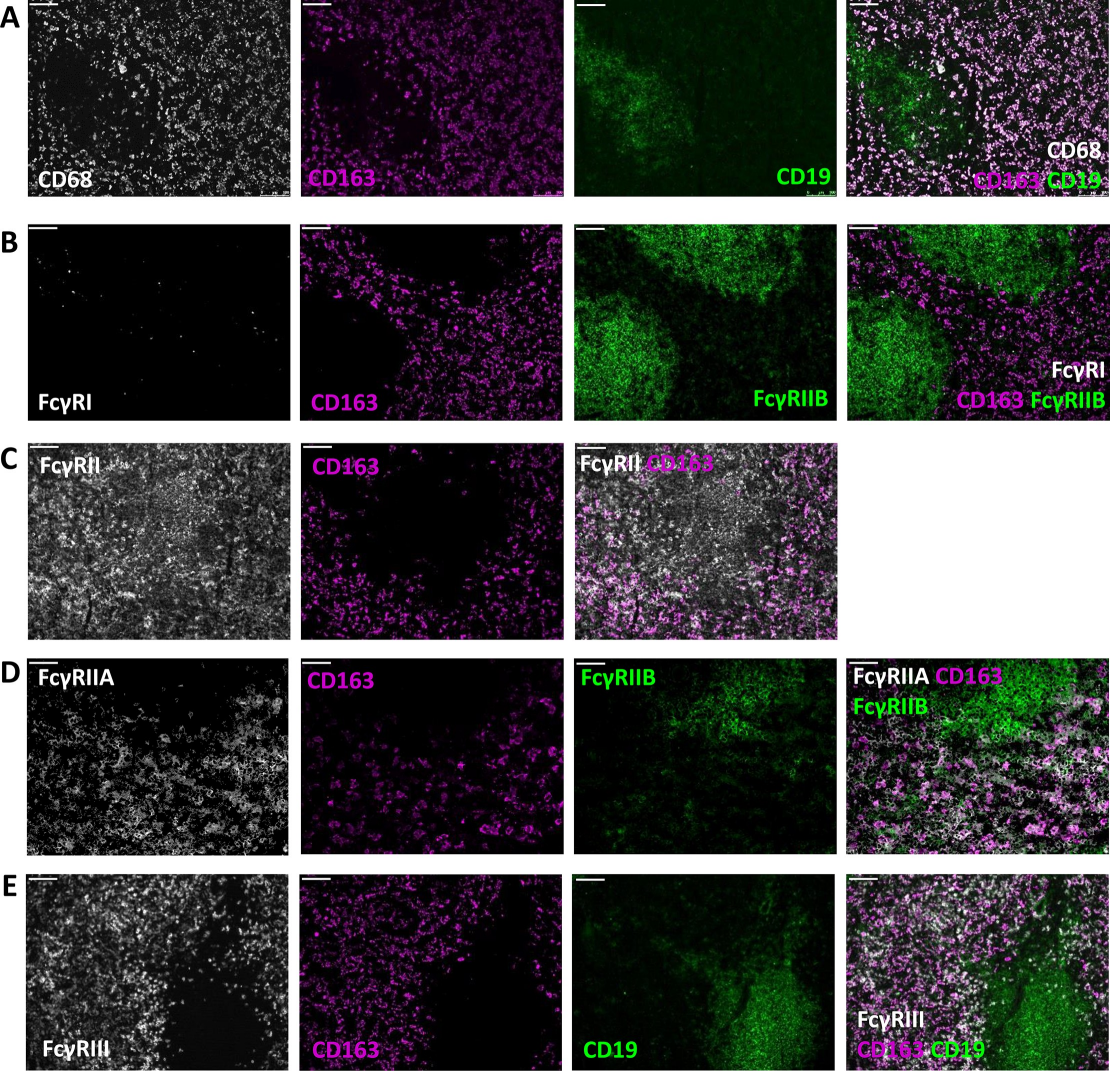
Red pulp macrophages express FcγRIIA, and FcγRIII. Immunofluorescence stainings of spleen sections with CD163 and (A) CD68 and CD19; (B) FcγRI and FcγRIIB/C; (C) FcγRII; (D) FcγRIIA and FcγRIIB/C (E) FcγRIII and CD19. Magnification 10x. Scale bars equal 100 μm. Figures are representative of n = 3 spleens from different donors. We have previously shown immunofluorescent stainings of human spleen tissue.^18^ Images in this figure are from sections from a different human spleen.

To investigate the FcγR expression pattern of tissue-resident macrophages, we performed co-stainings of macrophage markers and MoAbs specifically recognizing the different FcγR isoforms on sections of different tissues. Our study should be viewed with some limitations, because the different tissue samples were obtained from patients with various pathologies which were assumed to have no impact on FcγR expression, as indicated in the Methods section. Moreover, the tested tissues were not obtained from the same individual. Differences among individuals can thus not be excluded. We characterized human spleen red pulp macrophages by CD163 expression (Fig 1A). These macrophages expressed FcγRIIA and FcγRIIIA, but hardly if any FcγRI and FcγRIIB (Fig 1B–1E), as we have previously shown [18]. Marginal zone macrophages, characterized by the expression of CD169, were found to only express FcγRIIA [18].

**Fig 2:**
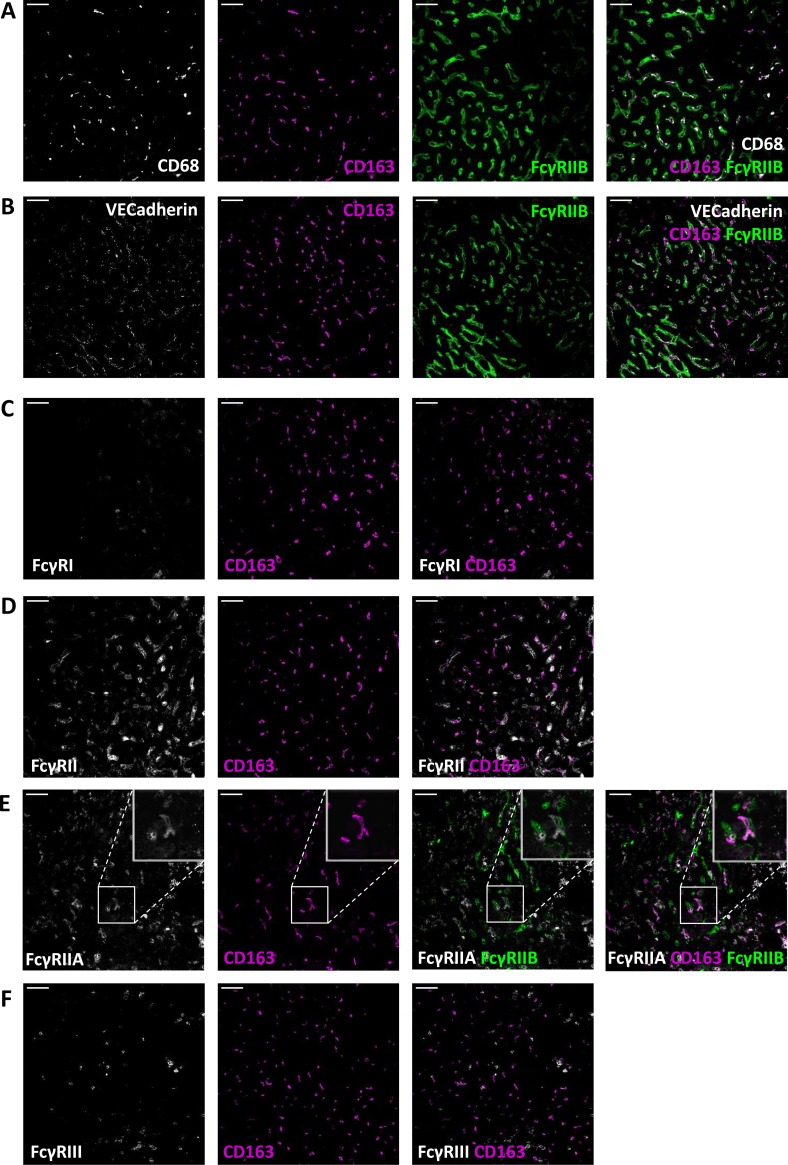
Kupffer cells express FcγRIIA, FcγRIIB and FcγRIII. Immunofluorescence stainings of liver sections with CD163 and (A) CD68 and FcγRIIB/C; (B) VECadherin and FcγRIIB/C; (C) FcγRI; (D) FcγRII; (E) FcγRIIA and FcγRIIB/C; (F) FcγRIII. Magnification 20x. Scale bars equal 50 μm. Figures are representative of n = 2 livers from different donors.

Immunostainings of liver sections showed that Kupffer cells, stained by the expression of the common macrophage marker CD68, were also positive for CD163 (Fig 2A). Subsequent stainings revealed that Kupffer cells express FcγRII (Fig 2D and 2E), FcγRIII (Fig 2F), and no if any FcγRI (Fig 2C). Apart from being positive on Kupffer cells, liver sections showed remarkably high expression of FcγRIIB on tissue cells that were clearly different from macrophages and enriched throughout the liver sections. These cells could be morphologically characterized as sinusoidal endothelial cells. Co-stainings of anti-VE-Cadherin, which marks endothelial cells, and MoAb 2B6, specifically recognizing FcγRIIB [22], confirmed the expression of FcγRIIB on sinusoidal endothelial cells (Fig 2B).

**Fig 3:**
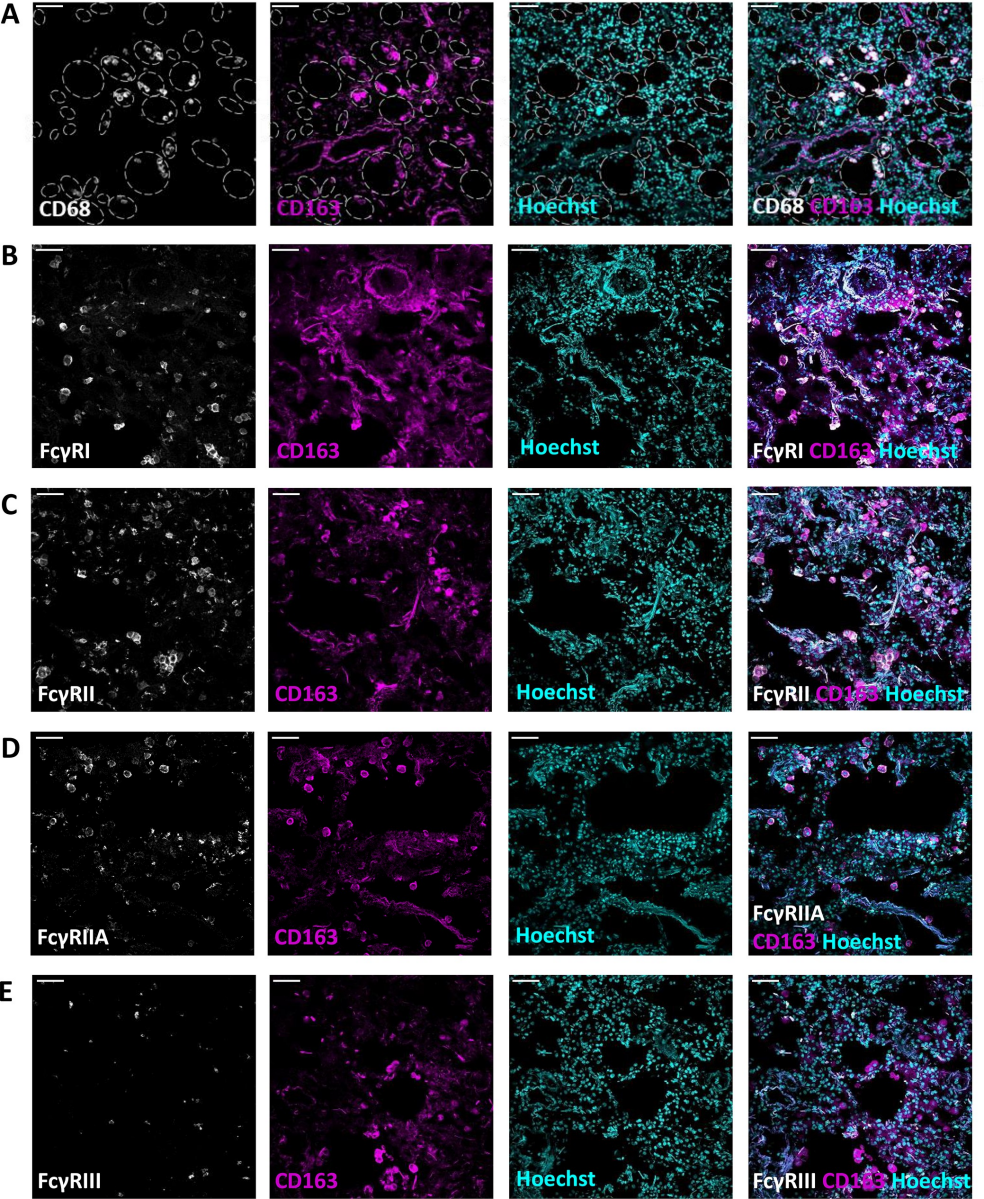
Lung macrophages express FcγRI and FcγRII. Immunofluorescence stainings of lung sections with CD163, Hoechst and (A) CD68; (B) VECadherin; (C) FcγRI; (D) FcγRII; (E) FcγRIIA; (F) FcγRIII. Magnification 20x. Scale bars equal 50 μm. Figures are representative of n = 3 lungs from different donors. In (A) alveolar spaces are indicated with grey dashed lines.

Lung sections were found to be highly autofluorescent. Immunostainings of lung sections revealed that alveolar macrophages were also characterized by the expression of CD163 and the common macrophage marker CD68 (Fig 3A). Stainings showed that alveolar macrophages expressed FcγRI (Fig 3B) and FcγRIIA (Fig 3C and 3D). Based on these tissue sections, the expression of FcγRIII seemed to be low on alveolar macrophages (Fig 3E).

### *Ex vivo* stainings of isolated macrophages

Because of the lack of accurate quantitation of FcγR expression and unavoidable background staining and autofluorescence, it is difficult to characterize the different tissue-resident macrophages in full detail based on immunostainings of tissue sections alone. For this reason and in order to compare the FcγR expression levels of macrophages from the different tissues with each other, we freshly isolated macrophages from the various tissues. By isolating the various macrophage subsets, we were able to *ex vivo* characterize these cells by cytospin and flow cytometry. Alveolar macrophages were obtained via a broncho-alveolar lavage and in this way, we studied these cells in more detail *ex vivo* by direct staining.

By cytospin we were able to analyze the morphology of the cells. Macrophages were easily detected by their round nucleus and granular bodies (S1 Fig). Macrophages that were cultured from blood monocytes in the presence of either GM-CSF or M-CSF were observed to be slightly bigger than tissue-resident macrophages by flow cytometry.

**Fig 4:**
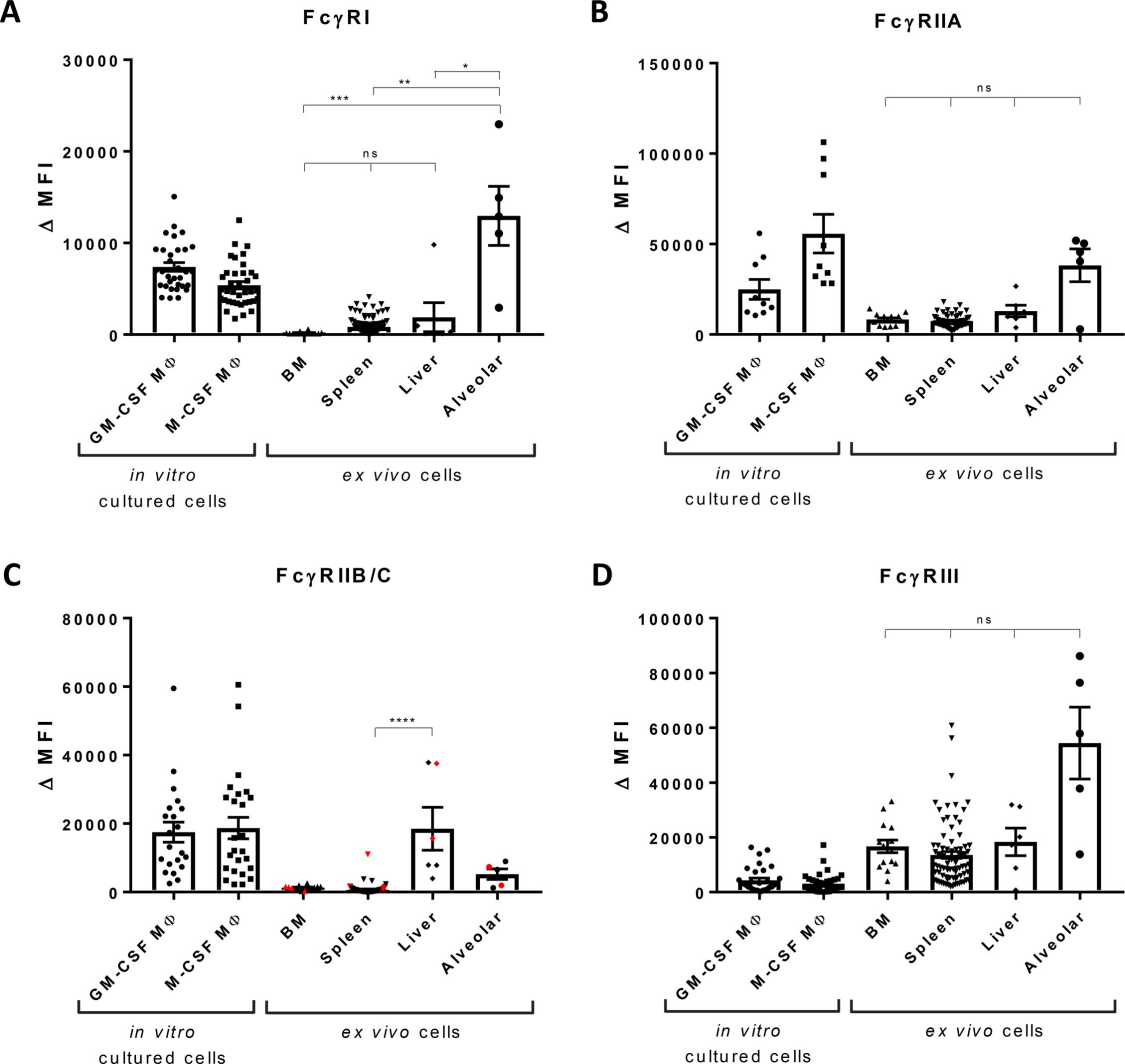
FcγR expression by macrophages from different tissues. Expression of (A) FcγRI, (B) FcγRIIA (MoAb IV.3), (C) FcγRIIB/C (MoAb 2B6), individuals encoding *FCGR2C*-ORF are indicated in red, and (D) FcγRIII by monocyte-derived macrophages cultured in the presence of GM-CSF, n = 9–32, and M-CSF, n = 9–39, by bone marrow macrophages, n = 14, red pulp macrophages, n = 83, Kupffer cells, n = 5, and alveolar macrophages, n = 5, determined by flow cytometry. For the cultured macrophages, individuals encoding *FCGR2C*-ORF were excluded from analysis. Data shown are median fluorescence intensity, corrected for the proper isotype control. For FcγRIIB/C expression, the levels on BM macrophages versus Kupffer cells from liver were nearly significant, p = 0.058. *p < 0.05, **p < 0.01, ***p < 0.001, ****p < 0.0001, ns = not significant.

The isolated tissue-resident macrophages were further characterized by flow cytometry to determine the FcγR expression pattern of MDMs and macrophages from the various tissues available, i.e. bone marrow, spleen, liver and lung (Fig 4; Tables 1 and 2; S2 Fig). We always found homogeneous expression of the different tested FcγRs for macrophages from a certain tissue, suggesting that they form a homogeneous cell population and do not represent macrophage subsets (S3 Fig). MDMs were cultured in the presence of either GM-CSF or M-CSF for nine days and expressed an FcγR pattern distinct from each other. GM-CSF macrophages mainly expressed FcγRI and FcγRIIIA, whereas M-CSF macrophages expressed more FcγRIIA, as we have previously published [2,3].

**Table 1:** Means and interquartile range (IQR) of FcγR expression levels by MDMs and tissue-resident macrophages, as shown in Fig 4A–4D.

	GM-CSF MΦ	M-CSF MΦ	BM	Spleen	Liver	Alveolar
FcγRI	7385 (3963)	5397 (3137)	164 (68)	832 (1238)	1891 (3072)	12954 (11963)
FcγRII (AT10)	43493 (17138)	119015 (48573)	11478 (5145)	10071 (4083)	24747 (21049)	69555 (66441)
FcγRIIA (IV.3)	24992 (28522)	55728 (62494)	8289 (5635)	7450 (4529)	12979 (11071)	38240 (29528)
FcγRIIB/C (2B6)	17505 (16701)	18710 (21615)	1269 (595)	729 (571)	18484 (30761)	5217 (6557)
FcγRIII	4358 (5785)	3060 (3957)	16722 (13586)	13574 (11217)	18332 (24659)	54430 (55550)

**Table 2:** Overview of FcγR expression by macrophages from BM, spleen, liver and lung.

	FcγRI	FcγRIIA	FcγRIIB	FcγRIII
**Bone marrow macrophages**	-	+	-	++
**Red pulp macrophages (spleen)**	+/-	+	-	++
**Kupffer cells (liver)**	+/-	+	+	++
**Alveolar macrophages (lung)**	++	+	-	++
**GM-CSF cultured macrophages**	++	+	+	+
**M-CSF cultured macrophages**	+	++	+	-

Bone marrow macrophages expressed no if any FcγRI (Fig 4A). Spleen and liver macrophages expressed a little more FcγRI, whereas alveolar macrophages expressed remarkably high levels of this receptor. FcγRI expression by alveolar macrophages reached expression levels that exceeded those of cultured macrophages. These findings were in line with the tissue section stainings, where alveolar macrophages were clearly shown to express FcγRI.

The expression of FcγRII was determined by staining with MoAb AT10, an antibody that is known to bind to all three isoforms FcγRIIA,B,C [22] (S2 Fig), whereas MoAb IV.3 most specifically recognizes FcγRIIA (Fig 4B). These stainings showed that FcγRIIA is expressed by macrophages from all different tissues. The expression of FcγRIIB, the only inhibitory IgG receptor, was specifically assessed by MoAb 2B6, which recognizes FcγRIIB/C [22]. By genotyping we determined which individuals have an *FCGR2C*-Open Reading Frame (ORF) and could therefore express FcγRIIC [20,21]. The presence of *FCGR2C*-ORF is indicated in red (Fig 4C). In case of an *FCGR2C*-STOP (indicated in black), no functional FcγRIIC can be expressed, in which case MoAb 2B6 specifically stains FcγRIIB. The inhibitory IgG receptor FcγRIIB was found to be expressed at a very low level if at all by most tissue macrophages except the Kupffer cells in the liver which–instead–demonstrated a significantly higher expression of FcγRIIB (Fig 4C).

Most remarkably, all tissue-resident macrophages, in contrast to MDMs, expressed FcγRIII (Fig 4D), at significantly higher levels than MDMs. We previously found the FcγRIII expression by red pulp macrophages to be specifically FcγRIIIA [18]. The expression level of FcγRIIIA correlates with *FCGR3A* copy number. For *FCGR1* and *FCGR2B* no copy number variation occurs. This can explain the bigger spread in the FcγRIIIA levels compared to FcγRI and FcγRIIB. A subpopulation of about 10% of the blood monocytes is known to express FcγRIIIA (CD16). These CD16^pos^ cells are believed to represent a specific subset of monocytes with a more mature phenotype than the CD16^neg^ monocytes [23,24]. Reasoning that these specific circulating monocytes could be a source of a fraction of the tissue-resident macrophages, we investigated whether these cells showed a different phenotype upon the standard nine days of culturing in either GM-CSF or M-CSF. Upon culturing into MDMs, the MACS sorted peripheral blood monocytes being either CD16^pos^ or CD16^neg^, expressed equal levels of this IgG receptor FcγRIIIA and of any of the other FcγRs (S4 Fig), which was already the case from day 3 of culturing. To exclude any specific effect of cell-cell interaction, we performed co-cultures of CD16^pos^ and CD16^neg^ monocytes and found the subsets of monocytes to form a homogeneous population.

## Discussion

Recently parabiotic and adoptive-transplant studies in rodents have shown that tissue-resident macrophages are derived from embryonic precursors and maintain themselves by self-renewal [6,16]. Most tissue-resident macrophages, except those in the intestine and skin, do not differentiate from peripheral blood monocytes, but are derived from non-hematopoietic embryonic precursors [4–8]. Whereas most functional studies about human macrophages are being performed with *in vitro* cultured MDMs, the M1 and M2 cultured macrophages represent extremes of a multidimensional spectrum [9,10,25]. To investigate *in vivo* human tissue-resident macrophages and compare these with MDMs, we isolated primary human macrophages from various tissues. Since macrophages play an important role in the uptake of immune complexes and show phagocytosis of IgG-opsonized particles via their FcγRs, we investigated the tissue-specific IgG receptor expression of human macrophages in detail.

Tissue-resident macrophages showed a different FcγR expression profile compared to MDMs. The biggest difference was found to consist of high expression of FcγRIII, CD16, which was much higher in case of tissue-resident macrophages of all tested tissues than for MDMs.

A distinct subset of blood monocytes, around 10%, expresses CD16 [23]. Previous studies have shown that these cells are phenotypically and functionally different from the CD16^neg^ monocytes and represent a more mature phenotype. For instance, phagocytosis of opsonized erythrocytes by CD16^pos^ monocytes has been reported to be less efficient than by CD16^neg^ monocytes [23,24]. It was also suggested that CD16^pos^ monocytes would resemble certain types of tissue-resident macrophages [24]. Here we report that upon culturing the two subpopulations of monocytes into MDMs, these cells form a homogenous cell population that after nine days expressed equal levels of FcγRIII, although still at a much lower level than was observed in tissue-resident macrophages. Thus, the original difference in FcγRIII expression on circulating monocytes entirely disappeared once the cells were differentiated into MDMs.

A noticeable difference among macrophages from different tissues is their expression of FcγRI. FcγRI expression was very low if present on bone marrow macrophages, while spleen and liver macrophages expressed intermediate levels of FcγRI and its expression was very high on alveolar macrophages. This can be explained because FcγRI can be rapidly upregulated upon cellular activation by interferon-γ (IFNγ) and granulocyte (monocyte) colony-stimulating factor (G(M)-CSF) [26,27]. Upregulation of FcγRI on neutrophils is used as marker for bacterial infections and systemic inflammation [26,28]. We previously reported the positive correlation between increased FcγRI expression on splenic neutrophils and red pulp macrophages [18]. We hypothesize that bone marrow macrophages do not express FcγRI, because they are quiescent and non-activated, while alveolar macrophages are constantly exposed to environmental triggers [10], which could easily explain their high FcγRI expression level.

The function of FcγRI on alveolar macrophages remains to be investigated. Because FcγRI is a high-affinity IgG receptor, it is thought to be saturated by IgG in plasma which prevents the binding of immune complexes [29]. There is IgG present in alveoli too, although about 1000 times less than in plasma, as measured in human BAL fluids of healthy individuals which had an IgG concentration of around 9 μg/mL [30,31]. Since the IgG concentration in alveoli is much lower than in plasma, we hypothesize that FcγRI is partially unoccupied, making it a true high-affinity receptor for IgG-opsonized microbial agents. This is in line with recent publications that show that FcγRI is not fully saturated by IgG, neither in mice [32] nor in humans [33], making it readily available for immune complexes.

Another possibility is that FcγRI is internalized by other tissue-resident macrophages, because it is bound by monomeric IgG, and can be rapidly upregulated on the surface after stimulation by immune complexes [34]. Because of the lower IgG concentration in alveoli, alveolar macrophages may not have their FcγRI internalized and for that reason have increased surface expression, but this explanation seems unlikely, since we could not detect FcγRI in red pulp macrophages or Kupffer cells which would be expected even when internalized. Moreover, RNA levels of *FCGR1* transcripts in red pulp macrophages are low, as we previously published [18], indicating that it is truly low expression and not due to immunofluorescence staining issues.

Another remarkable finding was that FcγRIIB, the only inhibitory receptor of the FcγR family, was highly expressed on sinusoidal endothelial cells in the liver. Previously, expression of FcγRIIB on sinusoidal endothelial cells was shown in mice and rats [35–39], but to the best of our knowledge this has never been clearly shown for humans. Whereas large immune complexes, consisting for example of antibody-opsonized bacteria or blood cells, were shown to be cleared by mononuclear phagocytes, small immune complexes were cleared by liver sinusoidal endothelial cells via the interaction with FcγRIIB [35]. Removal of immune complexes from the circulation by the inhibitory FcγRIIB may well be able to prevent the inflammatory consequences of their presence in the tissue upon spillage from the gut drained through the portal vein into the liver. Since the liver receives blood via the portal vein, which transports blood from the intestine, it is known to be continuously exposed to endotoxin [40]. The liver must dampen the inflammatory reaction in order to prevent systemic immune activation [16,41]. In this way the coexistence of Kupffer cells and sinusoidal endothelial cells in the portal sinusoids, their unique expression profile of FcγRs and their contribution to the clearance of immune complexes all contribute to homeostasis.

As mentioned in the Methods section, spleen and liver tissues were obtained from individuals undergoing surgery for unrelated reasons. Their hematopoietic compartment was therefore considered to be healthy. The alveolar macrophages were obtained by BAL for diagnostic purposes from patients suspected of COPD or sarcoidosis. These cells may be in a more inflamed state than cells from healthy individuals. We cannot exclude this may influence the FcγR expression levels. Moreover, it cannot be excluded that fixation of the tissue could influence FcγR staining efficacy to different degrees in different tissues.

Our study is a unique description of the FcγR expression pattern of primary macrophages in-situ and following their purification from different human tissues and–as far as we know–the first of its kind. Future functional experiments will demand more material to isolate enough cells to show the contribution of the different FcγRs in binding and uptake of IgG-opsonized particles.

Recent studies made use of transgenic or knock-in mice that express human FcγRs and studied the FcγR expression pattern of different cell types in blood and various tissues [42,43], including tumor-associated macrophages [44]. The FcγR expression pattern of blood leukocytes from these mice were compared to that of leukocytes from human blood. Although the FcγR expression patterns look similar between species, they are not identical. For instance, a lower FcγRIIA expression on mouse neutrophils and the presence of FcγRIIB on mouse monocytes, while this receptor is not expressed by human monocytes. This indicates that, although informative, FcγR expression by tissue macrophages obtained with transgenic or knock-in mice expressing human FcγRs, cannot be directly translated to humans.

The differences in FcγR expression profile between MDMs and tissue-resident macrophages, but also the differences between macrophages from the various tissues, raise the question whether these differences are due to the different origin of these cells or due to their milieu [16,45]. A recent study by Lavin et al. showed that the chromatin state of tissue-resident macrophages can partially be explained by their origin and partially by their environment. It is a combination of lineage-specific and tissue-specific factors that determines the identity and plasticity of tissue-resident macrophages [46]. Our study shows the remarkable differences between macrophages from different tissues and thereby highlights the diversity and complexity of this cell type.

In sum, we here show for the first time that human macrophages from bone marrow, spleen, liver and lung have unique FcγR expression patterns. We determined the expression patterns by *in vivo* characterization of stainings of tissue sections. Upon isolating macrophages for *ex vivo* characterization we investigated these cells in full detail. We showed that splenic red pulp macrophages express FcγRIIA and FcγRIII, Kupffer cells from the liver express FcγRIIA, FcγRIIB and FcγRIII. FcγRI was sometimes expressed to low extent. Alveolar macrophages express FcγRI, FcγRIIA and FcγRIII. We found the FcγR expression patterns of tissue-resident macrophages to be totally different from the expression patterns of *in vitro* cultured MDMs.

## Supporting information

S1 Fig Cytospins of macrophages. Cytospins of monocyte-derived macrophages cultured in the presence of (A) GM-CSF and (B) M-CSF, cytospins of tissue macrophages: (C) bone marrow, (D) sorted red pulp macrophages, (E) cells from liver and (F) cells from bronchoalveolar lavage. Magnification 50x. Figures are representative of at least n = 3 different donors.
(TIF)

S2 Fig FcγRII expression by macrophages from different tissues. Expression of FcγRIIA,B,C (MoAb AT10) by monocyte-derived macrophages cultured in the presence of GM-CSF, n = 32, and M-CSF, n = 39, by bone marrow macrophages, n = 14, red pulp macrophages, n = 83, Kupffer cells, n = 5, and alveolar macrophages, n = 5, determined by flow cytometry. Individuals encoding *FCGR2C*-ORF are indicated in red. Data shown are median fluorescence intensity (MFI), corrected for the proper isotype control (ΔMFI), the error bars represent SEM. *p < 0.05, **p < 0.01, ns = not significant.
(TIF)

S3 Fig FcγR expression of macrophages from human spleen show homogeneous cell populations. Flow cytometry stainings of anti-FcγRI (clone 10.1), anti-FcγRIIA (clone IV.3), anti-FcγRIIB (clone 2B6) and anti-FcγRIII (clone 3G8) of macrophages (upper panel) and monocytes (lower panel) isolated from human spleen and their appropriate isotype controls (upper rows). Median fluorescence intensity is shown on the x-axis. Data are from representative spleen sample.
(TIF)

S4 Fig Upon culturing into MDMs, the originally CD16^pos^ and CD16^neg^ monocytes show identical FcγR expression levels. FcγR expression by MDMs cultured in the presence of GM-CSF (left) and M-CSF (right) of originally CD16^pos^ monocytes (black) and CD16^neg^ monocytes (grey), n = 4. Data shown are median fluorescence intensity (MFI), corrected for the proper isotype control (ΔMFI), error bars represent SEM.
(TIF)
